# Sustainable Development and Performance Evaluation of Marble-Waste-Based Geopolymer Concrete

**DOI:** 10.3390/polym12091924

**Published:** 2020-08-26

**Authors:** Wei-Hao Lee, Kae-Long Lin, Ting-Hsuan Chang, Yung-Chin Ding, Ta-Wui Cheng

**Affiliations:** 1 Institute of Mineral Resources Engineering, National Taipei University of Technology, Taipei 10608, Taiwan; woohp44@gmail.com (T.-H.C.); ycding@ntut.edu.tw (Y.-C.D.); twchwng@ntut.edu.tw (T.-W.C.); 2 Department of Environmental Engineering, National I-Lan University, I-Lan 260007, Taiwan; kllin@niu.edu.tw

**Keywords:** geopolymers, geopolymerization, waste, marble, recycling, concrete, ready-mixed plant

## Abstract

The key objective of this study was to develop marble-based geopolymer concrete and examine the viability of its application as a sustainable structural material for the construction industry. The results of the research demonstrated that marble-based geopolymer concrete can be developed, and its physical/mechanical properties were shown to have a very good performance. According to various experimental tests and a large-scale ready-mixed plant test, it was found that the marble-based geopolymer concrete displayed a good workability and was not easily influenced by temperature changes. The results showed that marble-based geopolymer concrete has an excellent potential for further engineering development in the future.

## 1. Introduction

Recently, concrete scientists have been leaning toward saving energy, lowering the emissions of CO_2_, a primary greenhouse gas (GHG), into the atmosphere, with an eye toward controlling the heating of the Earth, and producing more durable, sustainable, user- and eco-friendly green construction materials that are preferably cost-effective too [1]. In recent times, the application of various solid wastes available in profuse quantities has been of key interest to engineers to manufacture novel construction composites, with a view toward getting rid of these hazardous wastes. Therefore, incorporating wastes to synthesize new construction composites is currently the most attractive topic for managing and reducing solid wastes and conserving restricted nonrenewable natural resources, irrespective of the production technology.

It is reported that producing one ton of ordinary Portland cement (OPC) via the present-day process not only consumes 1.7 tons of essential nonrenewable restricted resources of rocks and minerals [2,3] but also emits an almost equivalent quantity (about 0.85 tons) of anthropogenic CO_2_ into the atmosphere [4], which accounts for about 5% to 7% of all CO_2_ emissions [5]. CO_2_ alone is responsible for roughly 65% of global warming, and the cement industry is to blame for about 6% [6]. Provis and van Deventer [7] accounted for the next step: according to the prediction of the International Energy Agency (IEA), there will be around 9–10% (28 Gt.) of total CO_2_ emissions in the world by 2050. Regrettably, at present, no energy efficiency measures are sufficient for mitigation. The greenhouse effect is factor that is largely responsible for global warming. This is a matter of immense concern in terms of preserving pleasant environments, and it is also a warning signal for living communities on Earth [8].

According to the statistics of the Environmental Protection Agency (EPA) of Taiwan, 520.29 kg of carbon dioxide are emitted into the air for every ton of cement clinker produced [9]. Of note, according to the report, Taiwan’s CO_2_ emissions in 2018 reached 266.88 million metric tons [10]. On the other hand, there is plenty of waste that has diverse origins (such as marble waste (MW)), coming from a variety of sources; it creates landfill problems, resulting in health concerns and air pollution, driving climatic alterations and, in some places, affecting surface and sub-surface waters and water supplies.

Consequently, all of the above challenges have encouraged concrete scientists and researchers to seek alternative construction composites that are durable, sustainable, cost-effective, and user- and eco-friendly with a low carbon footprint and less burning of energy. Recently, geopolymer construction technology has emerged as a possible substitute for conventional construction systems. Geopolymers (GPs) belong to the category of novel inorganic polymers that are cementitious alumino-silicates, demonstrating an amorphous three-dimensional (3D) structure and made up of AlO_4_ and SiO_4_ tetrahedral units linked by shared oxygen atoms [11]. Geopolymerization is an exothermic reaction among precursors rich in alumina and silica of either industrial or geological origin with concentrated alkali activators in a combined solution of silicate and alkali hydroxide [7,12,13], at a temperature ranging from as low as ambient or even room temperature up to 100 °C and at an atmospheric pressure [14,15,16]. Geopolymeric composites demonstrate outstanding and unique characteristics in terms of durability, higher initial strength and mechanical properties; resistance to attack by chemicals like sulfates and acids; fire and thermal stability at elevated temperatures; exceptional resistance to freeze/thaw; anti-corrosion behavior; a carbon footprint that is nine times lower [16,17,18] and an energy use that is six times lower than current systems of OPC production [11]; little shrinkage; the ability to be cured by autoclave, etc., making them attractive alternatives to conventional systems [8,19,20]. All of these exceptional characteristics, as a whole, make them promising green structural materials for the future. Not only that, but GPs offer tremendous cost savings, in the range of 10% to 30%, as compared to the cost of conventional construction technologies [21].

Globally, an acceleration in the kinds and quantities of solid waste originating from industries, mining, agricultural and other domestic activities has posed a serious threat to the environment and ecology. In this regard, one of the chief industrial wastes comes from the marble industry and its mining processing stages in the form of sludge, powder or solid wastes. By and large, these wastes are disposed of on open land spaces, creating not only landfill predicaments but also environmental pollution. Furthermore, waste can be generated in the cladding or surrounding rock when the mine is being excavated in the form of a huge quantity of abandoned earth and stones. More often than not, the waste is dumped in any pit or vacant land in the vicinity. This leads to additional risks to the environment in the form of pollution through dust spreads covering a vast area. Particularly in dry conditions, the dust dries up and floats in the atmosphere, and it can deposit on vegetation, plants and crops, creating decaying ecological conditions for flora and fauna. Pure drinking water supply systems or water bodies can get contaminated. There are several ways to reuse marble waste for particular purposes, such as in the brick industry [22], transport infrastructure [23], cementitious material [24,25,26,27], geopolymer concrete [28], and aggregate or mineral additives [29]. Therefore, this research attempts to apply marble waste as a valuable construction material to geopolymer concrete manufacturing, thereby turning waste into wealth.

According to a report by the Taiwan Bureau of Mines, marble waste amounts to about 0.62 million tons annually in Taiwan [30]. This research work was planned in order to develop a novel marble-waste-based concrete at room temperature through the process of geopolymerization. Mine waste was sorted into coarse and fine materials, and they were recycled separately. Mine waste can be effectively resourced, so it can be applied to structural or nonstructural buildings without the high-temperature production steps and energy consumption of traditional cement production and related fields in which mine waste concrete can be applied and developed. Furthermore, this study explores the long-term durability and weathering ability of this novel green concrete. Since marble-based geopolymer concrete does not need to be fired at a high temperature, it can be manufactured at room temperature and can fully achieve the goals of energy saving and carbon reduction, as shown in this research. Thus, this research is actively involved in promoting eco-friendly technology in the building material sector.

## 2. Materials and Methods

### 2.1. Materials

The marble powder was collected from marble mine waste ground by a hammer mill (Taipei City, Taiwan), and its D_50_ was about 24.3 μm. The chemical composition analysis of marble powder is shown in Table 1. It was chiefly CaO, with a content of about 60.9%, and its loss on ignition was 35.2%. The mineral phase analysis results of marble by XRD (X-ray Diffraction) (BRUKER, Billerica, MA, USA) showed calcite.

The ground-granulated blast-furnace slag (GGBFS) powder (S4000) utilized for this study was provided by CHC Resources Co., Ltd. (Kaohsiung, Taiwan). The D_50_ of the GGBFS powder is 12.3 μm. The chemical composition analysis of the powder is shown in Table 1; it was mainly CaO, SiO_2_ and Al_2_O_3_, with the CaO content being the highest (about 57.9%). The mineral phase analysis of the GGBFS powder showed an amorphous phase.

The mineral wollastonite was employed to reduce the volume shrinkage powder in this study since it is needle-shaped. The particle size distribution range was 2.6–323.9 µm, and the D_50_ was 44 µm. The chemical composition analysis, showing mainly CaO and SiO_2_, is depicted in Table 1.

### 2.2. Alkali Solutions

Alkali solutions with various SiO_2_/Na_2_O and SiO_2_/Al_2_O_3_ molar ratios were prepared by mixing sodium silicate solution (9.5 wt % Na_2_O, 29 wt % SiO_2_), sodium hydroxide and sodium aluminate. The alkali solutions were prepared and ready to use the day before the experiment.

### 2.3. Geopolymer Concrete Preparation

Marble-based geopolymer concrete was synthesized by adding marble powder, GGBFS powder and wollastonite powder. After 3 min of pre-mixing, the mixture was then activated with an alkali solution with 1.28 or 1.5 SiO_2_/Na_2_O (with a controlled NaOH concentration of 4 and 6 M) and 50 SiO_2_/Al_2_O_3_ molar ratios at a 0.55 S/L ratio. After 10 min of thorough mixing, sand and gravel were added and blended for an additional 3 min to prepare geopolymer concrete with various geopolymer/sand/gravel ratios. Geopolymer concrete was then cast in Φ10 cm × 20 cm cylinder molds and left to cure at ambient temperature for three days. After three days, the geopolymer concrete was removed from the molds, and the samples were divided into two sections for indoor and outdoor curing until the testing date. For the indoor curing, the samples were placed in plastic containers, and the humidity was controlled at 90% at room temperature. For the outdoor curing, the samples were exposed to an uncontrolled outdoor environment on the roof of a five-story building in the center of Taipei City, Taiwan. Once cured for testing, the concrete specimens (Φ10 cm × 20 cm) were subjected to physical and mechanical property tests. The results were averaged from at least five samples for each test. The mix proportions of the marble-based geopolymer concrete are listed in Table 2.

## 3. Results

### 3.1. Effect of Powder Ratio and SiO_2_/Na_2_O Molar Ratio on Marble-Based Geopolymer Concrete

**Figure 1:**
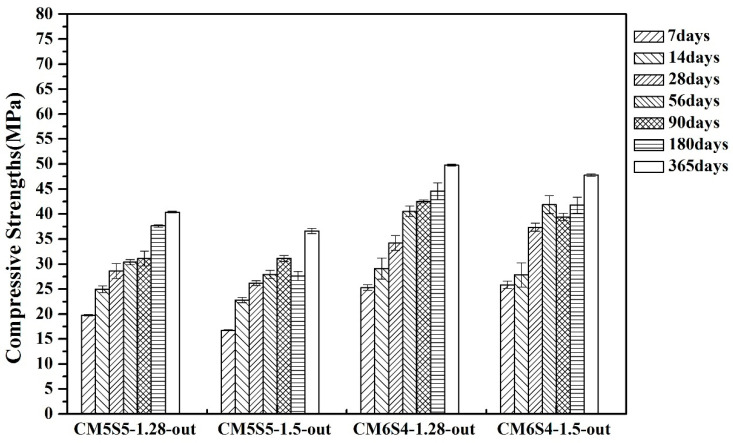
Effect of the powder ratio and SiO_2_/Na_2_O molar ratio on the compressive strength when curing in an uncontrolled outdoor environment.

The effect of the powder ratio and the SiO_2_/Na_2_O molar ratio on the compressive strength of marble-based geopolymer concrete curing in an uncontrolled outdoor environment, as described elsewhere [31], is illustrated in Figure 1. When the quantity of GGBFS increased, the compressive strength also increased. First, this is because the GGBFS powder itself is a highly reactive cementitious material, and second, its content was increased. In an alkaline environment, more calcium and silicon ions can be dissolved than marble. Consequently, the quantity of GGBFS added during the geopolymerization reaction contributes to the improvement of strength, but the original main objective of the research was to use marble powder for structural or nonstructural applications without involving the high-temperature production process of energy consumption. In this research, the proportion of marble and GGBFS powder was selected as 50:50.

The effect of the SiO_2_/Na_2_O molar ratios of 1.28 and 1.5 on the compressive strength when curing in an uncontrolled outdoor environment was also investigated. It was found that under the same powder conditions, the compressive strength of SiO_2_/Na_2_O with a molar ratio of 1.28 was better and reached 25–32 MPa over 28 days of curing. For long-term to 365-day curing, the compressive strength reached 40–50 MPa in the same case. The reason might be the SiO_2_/Na_2_O molar ratio of 1.5, which has increased sodium silicate. However, sodium silicate will dilute the alkalinity of the alkaline solution, resulting in less dissolution of the precursor gel during geopolymerization. The content is also higher, and it takes a long time to dehydrate. There are large amounts of micropores generated in the structure of the geopolymer that hinder the development of compressive strength, and no such phenomenon is found as the ratio is under the SiO_2_/Na_2_O ratio of 1.4 [32]. Therefore, the compressive strength of the test specimen prepared by using an alkaline solution of SiO_2_/Na_2_O with a molar ratio of 1.5 was poor in the long term; thus, for the subsequent experiments, we selected SiO_2_/Na_2_O with a molar ratio of 1.28.

### 3.2. Influence of Marble Ratio and Curing Environment on Marble-Based Geopolymer Concrete

A densified mixture design algorithm was used to design the proportion of marble-based geopolymer concrete ingredients [33,34]. The test was done with a cylindrical test specimen (10 cm diameter, 20 cm height) prepared with the CM5S5-DM and CM5S5-O experimental parameters, as presented in Table 3. In accordance with the experimental results, when the slurry content is high (CM5S5-O), the fluidity of the slurry can be improved to achieve a better slump and slump flow, and the slump flow can meet the requirements of the first-stage self-compacting concrete (ASTM C1611).

**Figure 2:**
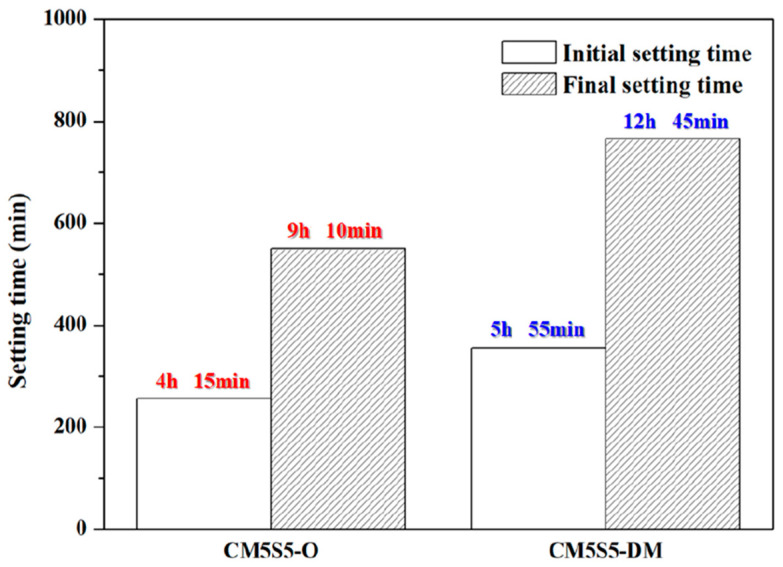
Setting time of marble-based geopolymer.

The initial and final setting times of marble-based geopolymer concrete were measured in accordance with the ASTM C403 standard. The effect of the binder/sand/coarse aggregate ratio on the hardening time of the concrete is displayed in Figure 2. Regarding the hardening time, when the slurry content is higher in a mixture, the calcium oxide content is also high, and calcium oxide shortens the hardening time. However, the operation time is still in line with current engineering requirements for more than 2 h.

**Figure 3:**
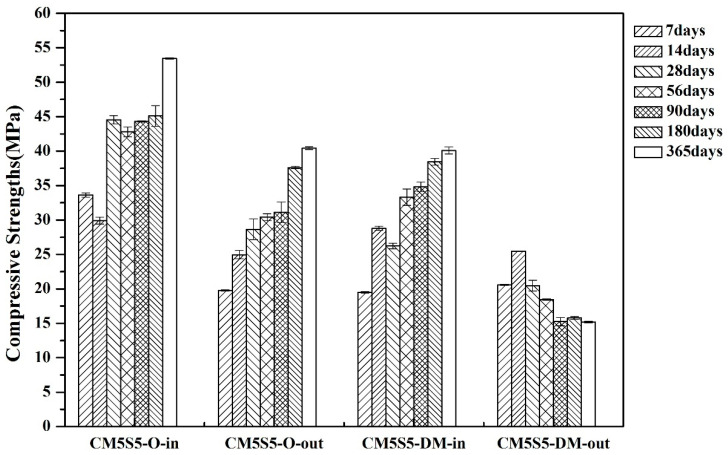
Effect of the binder ratio and curing environment on the compressive strength of marble-based geopolymer concrete under indoor and outdoor curing.

The effect of the binder ratio and curing environment on the compressive strength of marble-based geopolymer concrete under indoor and outdoor curing is shown in Figure 3. The experimental results demonstrated that the compressive strength with a lower binder content (CM5S5-DM) was lower than that of the slurry with a higher binder content (CM5S5-O), regardless of the indoor or outdoor curing conditions. Since the compressive strength of the concrete is derived from the binder, the compressive strength of the test specimen is also proportional to the binder content. Under uncontrolled outdoor curing conditions, after long-term weathering and with the increased curing age, the compressive strength of the sample with a lower binder content continued to decline. It was found that the marble-based geopolymer concrete with a higher binder content resisted the environmental conditions more.

**Figure 4:**
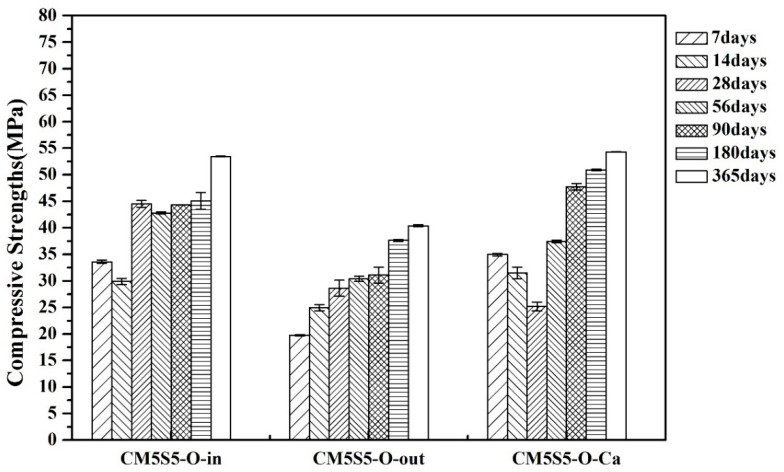
Effect of the curing environment on the compressive strength of marble-based geopolymer concrete (original designed ratio).

**Figure 5:**
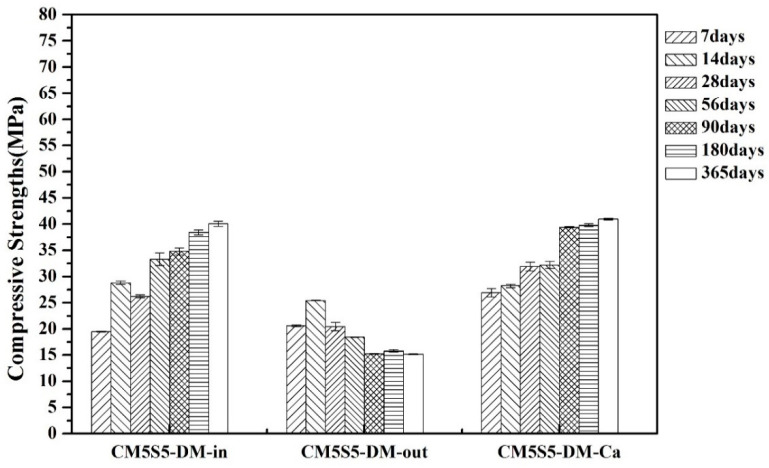
Effect of the curing environment on the compressive strength of marble-based geopolymer concrete (densified mixture designed ratio).

The effects of different curing environments on the compressive strength of marble-based geopolymer concrete are depicted in Figure 4 and Figure 5. The 28-day compressive strength while soaking the samples in lime water reached 25 and 32 MPa for the original designed ratio and the densified mixture designed ratio, respectively. Regardless of the ratio, the compressive strength after 365 days of saturation in lime water with long-term soaking increased, and with the original designed ratio it reached 54 MPa. Lime water curing has a stronger influence on marble-based geopolymer concrete, since the environment contributes saturated calcium and prevents the precipitation of calcium hydroxide in the concrete; therefore, it can have a more complete structure. The XRD results showed that the test specimen that was maintained in saturated lime water showed a higher content of gismondine than the indoor group did. The gismondine phase toward the C-A-S-H structure is the probable cause of the improved strength [31,35].

According to the experiment results shown, although the binder amount increase will affect the setting time of geopolymer concrete, the binder will also provide compressive strength to geopolymer concrete. Additionally, geopolymer concrete cannot be soaking in lime water because the Ca ions will react with free Si in the geopolymer, causing a decrease in strength. The above two phenomena are different from OPC concrete.

### 3.3. Effect of NaOH Molar Concentration on Marble-Based Geopolymer Concrete

In this test, marble-based geopolymer concrete was prepared by selecting the original designed ratio of the best parameters. Since the compressive strength with curing for 28 days can reach 44.5 MPa, the concentration of the alkali solution is reduced to achieve a low cost.

The effect of reducing the NaOH molar concentration to 4 M on the fresh properties of marble-based geopolymer concrete is shown in Table 4. Decreasing the concentration of alkaline solution has a small impact on the degree of the slump and slump flow; in terms of the hardening time, due to the reduced concentration, the alkali solution dissolves the surface of the powder, and the aluminum gel is relatively reduced, resulting in a slower geopolymerization.

**Figure 6:**
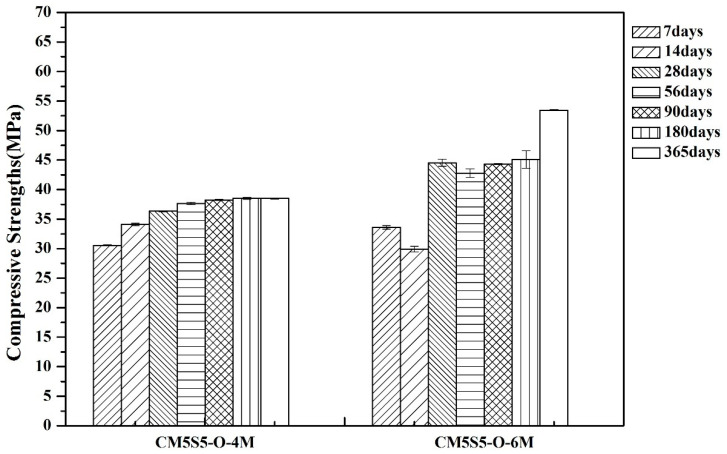
Effect of the NaOH molar concentration on the compressive strength of marble-based geopolymer concrete.

The effect of reducing the molar concentration on the compressive strength of marble-based geopolymer concrete is shown in Figure 6. On account of the reduced concentration, the alkaline solution makes the surface of the powder dissolve and there is relatively less aluminum gel, slowing down the inorganic geopolymerization reaction kinetics; therefore, the compressive strength tends to decrease slightly, but the compressive strength is stable at 28 days and at 365 days, at more than 30 MPa.

The effect of reducing the molar concentration on the shrinkage of marble-based geopolymer concrete is demonstrated in Table 5. The experimental results led us to note that because of the reduction of the molar concentration, the alkali solution somewhat reduced the dissolved silicon and aluminum gel from the powder surface; hence, the inorganic geopolymerization reaction was slower, and thus a low shrinkage rate was obtained. After 90 days of curing the mixture CM5S5-O-4M, the shrinkage rate was only −0.19%.

**Figure 7:**
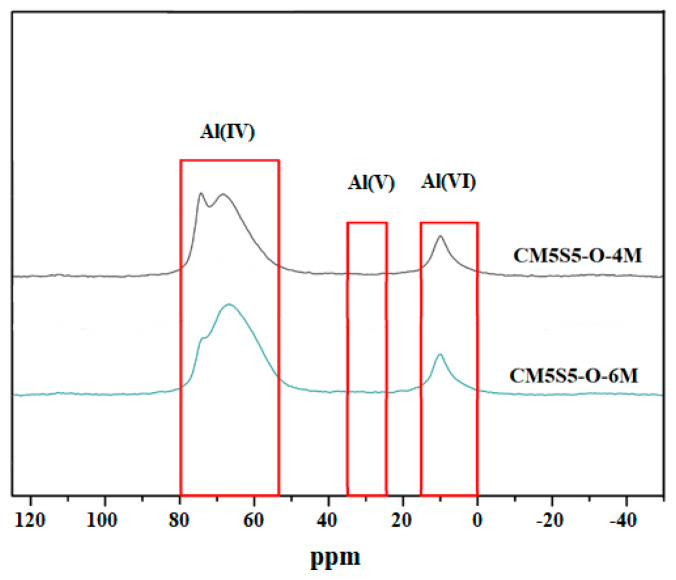
^27^Al NMR spectrum of marble-based geopolymer concrete.

^27^Al and ^29^Si NMR analyses were performed to identify the microstructure of marble-based geopolymer concrete using a Bruker 400 MHz Avance III NMR spectrometer. From the ^27^Al NMR spectrum, as shown in Figure 7, it can be found that the tetra-coordinated Al(4) atoms correspond to a chemical shift of 68–75 ppm, while the hexagonal Al(6) coordination exhibits a chemical shift of about 10 ppm. Both marble-based geopolymer concrete samples mainly contained Al(4) structures.

**Figure 8:**
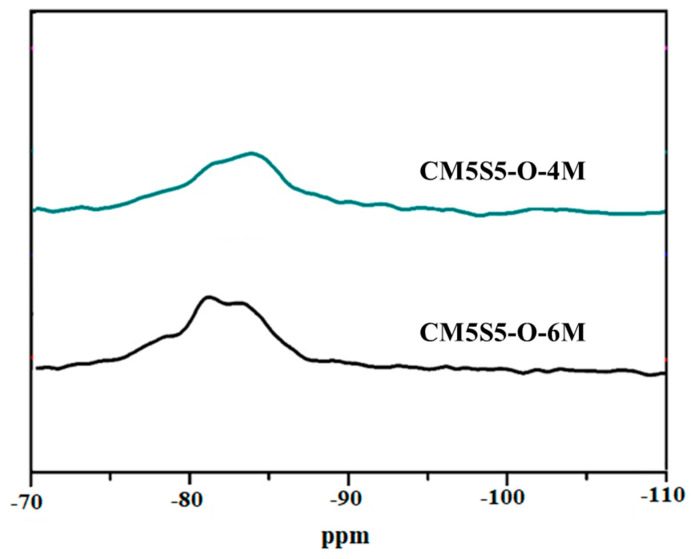
^29^Si NMR spectrum of marble-based geopolymer concrete.

Figure 8 shows the ^29^Si NMR spectrum of marble-based geopolymer concrete. A full range of siloxane units was found; as shown, the ^29^Si signal spans between −75 and −88 ppm in both samples [31]. Due to the broadening and overlapping of the ^29^Si signals, Peak Fit software, developed by Jandel Scientific, was used to calculate the relative deconvoluted peak areas of each individual ^29^Si unit. After curve fitting, the fraction area of the $Q_{4}^{4}$ silicon centers from the ^29^Si MAS NMR spectra of marble-based geopolymer concrete increased from 51.8% to 52.42% as the alkali solution concentration increased from 4 M to 6 M, as shown in Table 6. It is noted that the higher fraction area of $Q_{4}^{4}$ indicates a denser geopolymer structure [36]. Therefore, the compressive strength of CM5S5-O-6M also corresponds to the fraction of $Q_{4}^{4}$ and increases from 36 to 45 MPa after 180 days of curing.

### 3.4. Evaluation of Recycling Waste RC as Marble-Based Geopolymer Concrete Coarse Aggregate Additive

In this experimental study, marble-based geopolymer was synthesized by selecting the original designed ratio of the best parameters, and the feasibility of applying this concrete (CM5S5-WB) was discussed by utilizing recycled concrete aggregates in place of conventional coarse aggregates. The experimental findings show that the slump was satisfied; statistically, the slump was 280 mm and the slump flow was 690 mm. The effect of using recycled aggregate as a replacement for natural coarse aggregate on the hardening time of marble-based geopolymer concrete is shown in Table 7. The hardening time of the remaining recycled aggregate was shorter, since its calcium content was higher; hence, the hardening time was shortened. Another reason for this is that recycled concrete aggregates have high porosity and water absorption rates, reaching 24% and 10%, respectively. Similar results were also found elsewhere [37].

**Figure 9:**
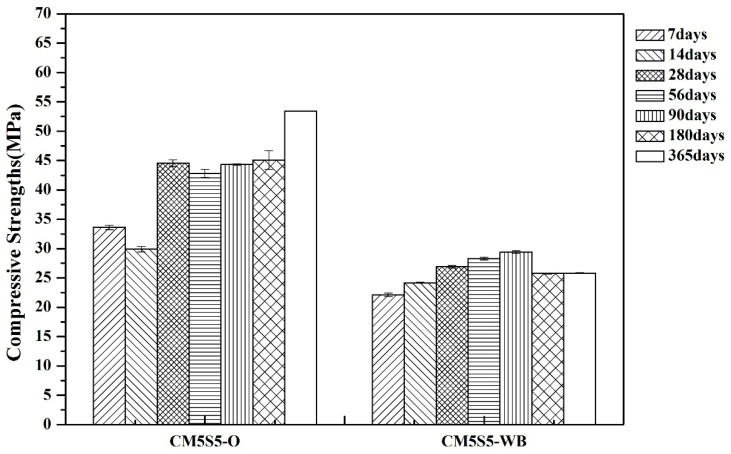
Compressive strength of marble-based geopolymer concrete produced with recycled aggregate.

The compressive strength of marble-based geopolymer concrete manufactured with recycled aggregates is shown in Figure 9. The compressive strength of the CM5S5-WB sample after seven days of curing reached 22 MPa or more, and over time it was found to increase slowly. Due to the low strength of recycled aggregate when compared to conventional coarse aggregate, the compressive strength of marble-based geopolymer concrete manufactured with recycled aggregate is lower, but it can still reach around 25 MPa.

The shrinkage rate of the marble-based geopolymer manufactured with recycled aggregate is shown in Table 8. Since recycled aggregate will absorb alkaline solution, the shrinkage rate is trivial.

### 3.5. Physical Properties of Test Parameters of Marble-Based Geopolymer Concrete

The test results of the physical properties of the test parameters are represented in Table 9 and Table 10. The test results show that, for the CM5S5-O-4M mix, the porosity and water absorption decreased and the bulk density increased with an increased curing age, which represents the continuous development of marble-based geopolymer concrete. Marble-based geopolymer concrete produced with recycled aggregate has a higher water absorption than the original designed concrete. The residual cement sticking to the outer surface of recycled aggregate will absorb alkali liquid. The pH test results showed that as the curing age increased, the pH had a tendency to decrease, and the pH was found to be under 11 after 365 days of curing. Therefore, marble-based geopolymer concrete may only be used in unreinforced concrete.

### 3.6. Large-Scale Test of Marble-Based Geopolymer Mortar in Ready-Mixed Plant

**Figure 10:**
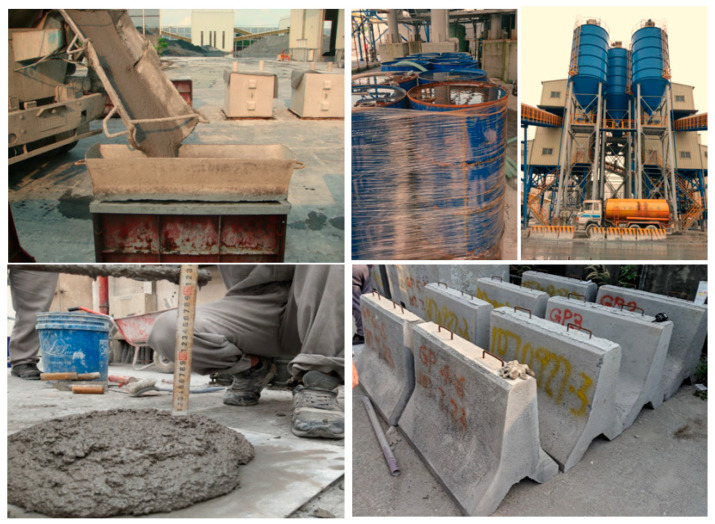
Samples at a ready-mixed plant.

The present research study is aimed at stabilizing marble waste by using innovative geopolymer technology as a matrix. Lab-scale experimental findings revealed that the compressive strength of marble-based geopolymer mortar could achieve a compressive strength of 42 MPa after 28 days. Several pilot-scale cubic meter marble-based geopolymer mortar blocks were developed in a ready-mixed plant (see Figure 10). The compressive strength demonstrated that geopolymer technology not only totally stabilizes marble waste production, but it also turns it into value-added products. Table 11 and Table 12 show the mix proportions for marble-based geopolymer mortar, and the fresh and hardened properties of the mortar, respectively.

## 4. Conclusions

This study reports the performance of marble-waste-based geopolymer concrete. Based on the results in the previous sections, some conclusions can be made:

(1) Compared to the densified mixture design ratio, the strength of the original-ratio marble-based geopolymer concrete increases with age, whether it is indoors or outdoors. Since compressive strength is derived from the binder, it is proportional to the binder content.
(2) The current best ratio is the original ratio (CM5S5-O), and the alkaline solution is preferably SiO_2_/Na_2_O with a molar ratio of 1.28, which gives the highest compressive strength of 40–50 MPa for 28-day or long-term 365-day curing.
(3) The workability of marble-based geopolymer concrete can meet the requirements of first-stage self-compacting concrete. The slump and slump flow can achieve 520 and 640 mm, respectively.
(4) The water absorption rate of recycled aggregate is higher than that of natural aggregate. Therefore, recycled aggregate absorbs more alkali solution than natural aggregate does, which causes the alkali liquid to not react completely with the powder, thus reducing the compressive strength and shortening the setting time.
(5) The large-scale test for marble-based geopolymer mortar in a ready-mixed plant was successful. The fresh properties were good for making several cubic meter blocks and Jersey barriers, and the compressive strength reached 43 MPa after 28 days of curing.

Although marble-based geopolymer concrete may not be used in reinforced concrete when compared with Portland cement, it still shows good potential for further engineering applications.

## Figures and Tables

**Table 1:** Powder chemical composition and particle size. GGBFS, ground-granulated blast-furnace slag.

	wt %	Marble Powder	GGBFS Powder	Wollastonite Powder
Composition				
CaO		60.9	57.9	58.1
SiO_2_		1	27.7	37.5
Al_2_O_3_		1.3	11.2	2.3
P_2_O_5_		1.6	–	–
Fe_2_O_3_		–	0.4	0.8
K_2_O		–	0.3	–
LOI		35.2	–	–
Particle size (D_50_) μm		24.3	12.3	26.6

**Table 2:** Mix proportions of the marble-based geopolymer concrete.

Item No. *	Alkali Solution (Molar Ratio)		Powder Ratio (wt. Ratio)	
	SiO_2_/Na_2_O	SiO_2_/Al_2_O_3_	Marble:GGBFS	Wollastonite (wt %)
CM5S5–1.28	1.28	50	5:5	5
CM5S5–1.5	1.5			
CM4S6–1.28	1.28	50	4:6	5
CM4S6–1.5	1.5			

* C, concrete; M, marble; S, GGBFS.

**Table 3:** Fresh properties of marble-based geopolymer concrete.

Item No. *	Binder/Sand/Aggregate	Slump (mm)	Slump Flow (mm)
CM5S5-O	1:2.3:2.2	270	640
CM5S5-DM	1:3.1:3.4	240	520

* C, concrete; M, marble; S, GGBFS; O, original design; DM, densified mixture design algorithm.

**Table 4:** Effect of the NaOH molar concentration on the fresh properties of marble-based geopolymer concrete.

Item No.	NaOH Concentration	Mixture Proportion (wt. Ratio)	Slump (mm)	Slump Flow (mm)	Initial Setting Time	Final Setting Time
CM5S5-O-4M	4 M	1:2.3:2.2	270	650	5 h 10 min	12 h 15 min
CM5S5-O-6M	6 M	1:2.3:2.2	270	640	4 h 15 min	9 h 10 min

**Table 5:** Effect of a reduced NaOH molar concentration on shrinkage of marble-based geopolymer concrete.

Item No.	Shrinkage Rate (%)				
	7 Days	14 Days	28 Days	56 Days	90 Days
CM5S5-O-4M	−0.07	−0.14	−0.17	−0.19	−0.19
CM5S5-O-6M	−0.32	−0.38	−0.56	−0.58	−0.58

**Table 6:** Fraction area of silicon sites after curve fitting.

Item No.	Fraction Area (%)				
	*Q*_1_ and *Q*_2_	$Q_{4}^{4}$	$Q_{4}^{3}$	$Q_{4}^{2}$	$Q_{4}^{1}$
CM5S5-O-4M	38.84	51.8	7.39	1.61	0.36
CM5S5-O-6M	35.53	52.42	10.89	1.16	0

**Table 7:** Effect of recycled aggregate on the fresh properties of marble-based geopolymer concrete.

Item No.	Mixture Proportion	Slump (mm)	Slump Flow (mm)	Initial Setting Time	Final Setting Time
CM5S5-O	1:2.3:2.2	270	640	4 h 15 min	9 h 10 min
CM5S5-WB	1:2.3:2.2	280	690	3 h 20 min	8 h 10 min

**Table 8:** Shrinkage rate of marble-based geopolymer concrete synthesized with recycled aggregate.

Item No.	Shrinkage Rate				
	7 Days	14 Days	28 Days	56 Days	90 Days
CM5S5-O	−0.32	−0.38	−0.56	−0.58	−0.58
CM5S5-WB	−0.14	−0.24	−0.27	−0.31	−0.37

**Table 9:** Test results of the bulk density, porosity and water absorption rate of marble-based geopolymer concrete.

Item No.	Bulk Density (kg/m^3^)			Porosity (%)			Water Absorption Rate (%)		
	7 Days	14 Days	28 Days	7 Days	14 Days	28 Days	7 Days	14 Days	28 Days
CM5S5-O-6M	2230	2208	2100	8.0	7.9	8.4	18.0	17.2	19.2
CM5S5-O-4M	1950	2044	2111	11.4	11.8	9.8	22.3	24.2	20.6
CM5S5-DM	2240	2242	2160	20.0	16.1	20.0	8.0	7.2	9.0
CM5S5-WB	1886	1849	1855	28.4	28.9	28.9	15.1	15.7	15.6

**Table 10:** pH values of marble-based geopolymer concrete.

Item No.	pH Value						
	7 Days	14 Days	28 Days	56 Days	90 Days	180 Days	365 Days
CM5S5-O	12.2	11.9	11.8	11.4	11.2	11.0	10.8
CM5S5-DM	12.1	11.8	11.7	11.2	10.9	10.7	10.4

**Table 11:** Mix proportions of marble-based geopolymer mortar for making Jersey barriers at a ready-mixed plant.

Volume	L/S	Binder/Aggregate (wt. Ratio)	Proportion (kg)							Total Weight (kg)
			Alkali Solution	Marble Powder	GGBFS	Fly Ash	Fine Aggregate	Aggregate Water Content	Additional Water	
1.5 m^3^	0.4	1:2.788	315	180	400	200	2175	5.0%	226.5	3497

**Table 12:** Fresh and hardened properties of marble-based geopolymer mortar for making Jersey barriers at a ready-mixed plant.

Slump (mm)	Slump Flow (mm)	Compressive Strength (MPa)	
		7 Days	28 Days
210	310 × 320	28.7	43.0
